# Needs Assessment for Supports to Promote Pediatric Clinical Research Using an Online Survey of the Japanese Children’s Hospitals Association

**DOI:** 10.31662/jmaj.2019-0037

**Published:** 2020-04-07

**Authors:** Osamu Nomura, Toru Kobayashi, Chie Nagata, Takeshi Kuriyama, Mayumi Sako, Kazuyuki Saito, Akira Ishiguro

**Affiliations:** 1 Department of Education for Clinical Research, National Center for Child Health and Development, Tokyo, Japan; 2 Department of Management and Strategy, National Center for Child Health and Development, Tokyo, Japan; 3 Department of Clinical Research Promotion, National Center for Child Health and Development, Tokyo, Japan; 4 Center for Clinical Research, National Center for Child Health and Development, Tokyo, Japan; 5 Center for Postgraduate Education and Training, National Center for Child Health and Development, Tokyo, Japan

**Keywords:** clinical research, clinical trial, education, pediatrics

## Abstract

**Introduction:**

Infrastructure and the capacity to conduct clinical research in pediatrics have not been fully established in Japan. To elucidate the physicians’ perspectives on clinical research, level of experience, existing barriers, and requests for support, we conducted a survey at 34 children’s hospitals in Japan.

**Methods:**

In January 2016, an online survey with 13 questions was sent to approximately 2000 physicians working in 34 pediatric hospitals belonging to the Japanese Association of Children’s Hospitals and Related Institutions.

**Results:**

Of the 360 respondents, 318 (88.3%) had presentations at academic conferences, and 261 (72.5%) had publications in academic journals, in the previous year. The most common study designs of clinical research conducted were case reports and case series. The most requested supports were for statistical analysis, followed by study design, grant application, and English-language editing. Younger physicians were more likely to prefer educational lectures (*p *< 0.001), whereas experienced physicians were more likely to request support for conducting statistical analysis (*p *= 0.002). Whereas physicians who had ever led a clinical trial requested support for the development of study protocol (*p *= .013), those without this experience preferred support for literature review (*p *= .002) and consultation services for study design (*p *= .027).

**Conclusions:**

The requests for supports were different, depending on the physicians’ years after graduation and experience level in clinical research. In order to enhance clinical research in pediatrics, it is essential to provide appropriate types and levels of educational and support programs.

## Introduction

Clinical research promotion is vital to assist clinical decision-making by physicians and to improve the quality of health care. The evidence provided by clinical research has been incorporated into the development of new treatments, implementation of clinical practice guidelines, and health-related policymaking ^(1)^. In pediatrics, special attention should be paid to the conduct of clinical research due to the limited age population and unique requirements, such as informed assents and consents ^(2)^. Therefore, resource allocations for the promotion of pediatric research need to be made by taking into account the pediatrics-specific context ^(3)^.

Clinical research in all pediatric specialties has created an enormous knowledge base that has enhanced evidence-based pediatric care and policymaking for children worldwide ^(4), (5)^. However, infrastructure and the capacity to conduct clinical research in pediatrics have not been fully established in Japan. In 2014, a nationwide survey involving 31 children’s hospitals belonging to the Japanese Association of Children’s Hospitals and Related Institutions (JACHRI) was conducted to assess the needs for clinical research promotion ^(6)^. By analyzing the responses from the representatives of each institution, the needs for appropriate human resource allocation to address insufficient support and education were identified. Although this survey has contributed by describing the issues in clinical research promotion for pediatrics from the institutional perspective, it did not reflect the perceptions of researchers that may be related to academic activity at the individualized level. It is therefore necessary to reveal the individual needs of physicians when conducting clinical research at children’s hospitals. We conducted a survey at 34 children’s hospitals in Japan to understand the physician’s perspectives on clinical research, their levels of experience, and their requests for support.

## Materials and Methods

### Study design

We conducted a cross-sectional questionnaire survey to evaluate the needs for support for pediatric clinical research in Japan in 2016. We hypothesized that physicians’ needs for research support would vary, depending on their backgrounds, such as the number of clinical years and their research experience.

### Participants

Approximately 2000 physicians working at 34 hospitals that were members of the JACHRI were potentially eligible for participation in this study. We communicated with the contact personnel at each hospital, who then sent an email asking to fill out the online survey to the physicians working for that hospital. The eligible participants included not only pediatricians but also any physicians from other disciplines collaborating in pediatric care, such as pediatric surgeons at the JACHRI hospitals.

### Data collection

An online questionnaire survey using SurveyMonkey (www.surveymonkey.com) was conducted in January 2016. The survey included 13 items asking about the demographics of participants; experience in academic activities, such as academic presentations and journal publications; perspectives on conducting clinical research; experience in conducting clinical research; needs for support for academic activities; and satisfaction about the present support. In particular, we asked the participants regarding their experiences in academic activities such as giving research presentations as the first presenter at academic conferences and their publication experiences of research papers in academic journals, including peer-reviewed and non-peer-reviewed journals, regardless of the language and type of publication, but excluding abstracts, in the previous year.

### Data analysis

First, we summarized the respondents’ demographics and their answers to the questions and described the categorical variables as numbers and percentages. The respondents were categorized into four groups, depending on their years after graduation from medical school (≤5 years, 6 to 15 years, 16 to 25 years, ≥26 years), and Fisher’s exact tests were conducted to compare their perceptions regarding supports that would promote their academic activities based on their categorized years after graduation from medical school. In addition, the respondents were subdivided into those who ever led clinical trials and those who never led clinical trials, and again, we compared the participants’ perceptions regarding supports that would promote their academic activities among the different age (i.e., ≤ 5, 6–15, 16–25, and ≥26 years) groups. Data were analyzed using SAS 9.4 (SAS Institute, Cary, North Carolina, USA).

### Ethics

This study was approved by the Ethics Committee of the National Center for Child Health and Development (NCCHD) (ID: 2159).

## Results

Of approximately 2000 eligible physicians, 360 at 27 hospitals registered in or collaborating with the JACHRI responded to our online survey (response rate: 18.0%). As presented in Table 1, the distribution of participants’ years after graduation was as follows: ≤5 years, 5.6%; 6 to 15 years, 38.1%; 16 to 25 years, 31.1%; and ≥26 years, 25.3%. One hundred fifty-four (42.8%) of the respondents were satisfied with the clinical research supports provided by their institutions. Of all, 318 (83.3%) had one or more presentations at academic conferences, and 261 (72.5%) had some type of publication in academic journals in the previous year. In terms of experience in conducting clinical research, 292 (81.1%) had experience in some type of clinical research; case reports and case series and single-institute retrospective observational studies were the most frequently conducted types of studies. The participants reported that statistical analysis, research funding, and English editing were the common barriers to academic activities, that is, presentations at academic conferences and publications in academic journals (Table 2). With regard to the education and supports that the participants thought would promote their academic activities, supports for conducting statistical analysis, study design consultations, and support to obtain research grants were most frequently reported (Table 3). In terms of the relationship between the support needs and participants’ backgrounds (Table 4), although younger participants sought the opportunities for educational lectures (*p* < .001) and practical training using educational materials and software (*p* = .049), experienced physicians preferred to receive support for conducting statistical analysis (*p* = .002). From the aspect of research experience (Table 5), physicians who had ever led clinical trials requested support for study protocol development (*p* = .013) and statistical analysis planning (*p* = .022). Contrarily, physicians who had never led clinical trials preferred support for literature review (*p* = .002), consultation services for study design (*p* = .027), and educational lectures (*p* = .027).

**Table 1.** Demographics of Respondents, Academic Activities, and Clinical Research Experience (n = 360).

	Number (%)
Years after graduation from medical school, n (%)	
≤5 years	20 (5.6)
6 to 15 years	137 (38.1)
16 to 25 years	112 (31.1)
≥26 years	91 (25.3)
Satisfied with the current research support	154 (42.8)
Presentations at academic conferences as a first presenter in the previous year, n (%)	
None	42 (11.7)
1 or 2 presentations	144 (40.0)
3 or 4 presentations	100 (27.8)
5 to 9 presentations	65 (18.1)
≥10 presentations	9 (2.5)
Publications in academic journals in the previous year, n (%)	
None	99 (27.5)
1 or 2 publications	118 (32.8)
3 or 4 publications	60 (16.7)
5 to 9 publications	50 (13.9)
≥10 publications	33 (9.2)
Experience in clinical research	
None, n (%)	68 (18.9)
Any experience in leading clinical research, n (%)	292 (81.1)
Types of clinical research*	
Case report or case series report, n (%)	237 (65.8)
Single-institute retrospective observational study, n (%)	228 (63.3)
Single-institute prospective observational study, n (%)	82 (22.8)
Multi-institute retrospective observational study, n (%)	82 (22.8)
Single-institute interventional study, n (%)	55 (15.3)
Multi-institute prospective observational study, n (%)	53 (14.7)
Multi-institute interventional study, n (%)	38 (10.6)
Systematic review, n (%)	22 (6.1)

^*^Participants were asked to respond about all types of research they have conducted.

**Table 2.** Barriers to Academic Activities, Such as Presentations at Academic Conferences and Publications in Academic Journals^*^ (n = 360).

	n (%)
Statistical analysis	271 (75.3)
Research funding	185 (51.4)
English editing	155 (43.1)
Database creation	127 (35.3)
Ethics committee	106 (29.4)
Drafting of study protocol	98 (27.2)
Literature review	83 (23.1)
Identification of clinical questions	83 (23.1)
Data collection	75 (20.8)
Defining of research questions	66 (18.3)
Creation of tables and figures	36 (10.0)
Others	32 (8.9)

^*^Participants were asked to respond about all possible barriers for conducting academic activities.

**Table 3.** Supports that Would Promote Academic Activities^*^ (n = 360).

	n (%)
Support for conducting statistical analysis	193 (53.6)
Study design consultations	145 (40.3)
Support to obtain research grants	119 (33.1)
English editing	107 (29.7)
Support for statistical analysis planning	67 (18.6)
Support for ethics committee application	57 (15.8)
Support for study protocol development	56 (15.6)
Educational lectures	50 (13.9)
Support for data collection	49 (13.6)
Support for patient registration	38 (10.6)
Support for literature review	36 (10.0)
Practical training using educational materials and software	25 (6.9)
Support for the development of patient information sheet and informed consent form	21 (5.8)
Support for intellectual property and collaborative research	14 (3.9)
Support for pharmaceutical affairs	10 (2.8)
Others	21 (5.8)

^*^Participants were asked to select up to three possible support options.

**Table 4.** Relationship between Participants’ Perceptions of Supports that Promote Academic Activities and Years after Graduation from Medical School.

	≤5 years (n = 20)	6 to 15 years (n = 137)	16 to 25 years (n = 112)	≥26 years (n = 91)	*p*
Support for conducting statistical analysis, n (%)	4 (20.0)	66 (48.2)	69 (61.6)	54 (59.3)	.002
Study design consultations, n (%)	8 (40.0)	61 (44.5)	47 (42.0)	29 (31.9)	.275
Support to obtain research grants, n (%)	3 (15.0)	43 (31.4)	39 (34.8)	34 (37.4)	.256
English editing, n (%)	6 (30.0)	47 (34.3)	33 (29.5)	21 (23.1)	.343
Support for statistical analysis planning, n (%)	5 (25.0)	23 (16.8)	21 (18.8)	18 (19.8)	.762
Support for ethics committee application, n (%)	1 (5.0)	18 (13.1)	22 (19.6)	16 (17.6)	.288
Support for study protocol development, n (%)	4 (20.0)	13 (9.5)	19 (17.0)	20 (22.0)	.050
Educational lectures, n (%)	6 (30.0)	32 (23.4)	7 (6.3)	5 (5.5)	<.001
Support for data collection, n (%)	2 (10.0)	19 (13.9)	14 (12.5)	14 (15.4)	.923
Support for patient registration, n (%)	3 (15.0)	16 (11.7)	14 (12.5)	5 (5.5)	.251
Support for literature review, n (%)	3 (15.0)	14 (10.2)	8 (7.1)	11 (12.1)	.508
Practical training using educational materials and software, n (%)	4 (20.0)	12 (8.8)	6 (5.4)	3 (3.3)	.049
Support for the development of patient information sheet and informed consent form, n (%)	2 (10.0)	7 (5.1)	7 (6.3)	5 (5.5)	.769
Support for intellectual property and collaborative research, n (%)	0 (0)	5 (3.6)	4 (3.6)	5 (5.5)	.817
Support for pharmaceutical affairs, n (%)	0 (0)	5 (3.6)	2 (1.8)	3 (3.3)	.841

**Table 5.** Relationship between Participants’ Perceptions of Supports that Promote Academic Activities and Participants’ Experience in Leading Clinical Trials.

	Ever led clinical trials (n = 79)	Never led clinical trials (n = 281)	*p*
Support for conducting statistical analysis, n (%)	45 (57.0)	148 (52.7)	.525
Study design consultations, n (%)	23 (29.1)	122 (43.4)	.027
Support to obtain research grants, n (%)	31 (39.2)	88 (31.3)	.223
English editing, n (%)	19 (24.1)	88 (31.3)	.265
Support for statistical analysis planning, n (%)	22 (27.8)	45 (16.0)	.022
Support for ethics committee application, n (%)	14 (17.7)	43 (15.3)	.603
Support for study protocol development, n (%)	20 (25.3)	36 (12.8)	.013
Educational lecture, n (%)	5 (6.3)	45 (16.0)	.027
Support for data collection, n (%)	11 (13.9)	38 (13.5)	1.000
Support for patient registration, n (%)	8 (10.1)	30 (10.7)	1.000
Support for literature review, n (%)	1 (1.3)	35 (12.5)	.002
Practical training using educational materials and software, n (%)	3 (3.8)	22 (7.8)	.316
Support for the development of patient information sheet and informed consent form, n (%)	5 (6.3)	16 (5.7)	.789
Support for intellectual property and collaborative research, n (%)	5 (6.3)	9 (3.2)	.200
Support for pharmaceutical affairs, n (%)	4 (5.1)	6 (2.1)	.236

## Discussion

To our knowledge, this is the first study assessing Japanese pediatricians’ requests for supports in clinical research and academic activities. We newly found that their requests were different, depending on their backgrounds, including years after graduation and experience in conducting clinical trials. Specifically, the younger physicians and physicians without experience in clinical trial requested “basic” support, such as educational lectures and practical training using educational materials and software, and supports needed in the early stages of research, such as literature review and study design. The experienced physicians requested advanced supports for actually “conducting” clinical research, such as study protocol development and statistical analysis.

A nationwide study investigating Japanese pediatric residents’ research activities reported that the publications of the residents were mostly limited to case reports ^(7)^, indicating that younger Japanese physicians do not have much experience in conducting clinical trials. Therefore, as our respondents reported, it is reasonable to provide younger physicians with supports to develop their basic knowledge and skills for planning and conducting clinical research. We also found that advanced supports (e.g., statistical analysis) are required to promote experienced physicians’ research activities. Although it is important for experienced physicians to develop research skills to conduct clinical trials, careful mentorship for them is also critical to complete the trials and publish the results ^(8)^. In the United States, scholars systematically established nationwide clinical research mentorship programs along with defining the competencies of effective research mentorship and developing a measurement tool to evaluate the performance of research mentors ^(9), (10), (11), (12)^. However, there is a disparity in the quality and quantity of research mentorship among hospitals, depending on the characteristics of children’s hospitals in Japan ^(6)^. To address this gap, the competency of clinical research mentors first needs to be defined in the context of Japanese pediatrics.

We noted the differences in the requested supports for academic activities between experts and novices in the context of pediatric clinical research. In response to the “generational gap,” clinical research curriculum developers need to consider the antecedents of the gap, including age, clinical experience, and cultural variations ^(13), (14)^. Recent literature has emphasized that clinical research educators should pay attention to the differences in value systems among different generations of physicians to foster cross-generational relationships and to achieve effective teaching in clinical medicine ^(15), (16)^. In this vein, the results of our needs assessment demonstrated that “one size does not fit all” in developing an educational support system for pediatric clinical research. Rather, diverse and individualized curricula and resources are more effective in promoting Japanese physicians’ clinical research activities. The different support needs corresponding to the different levels of research experience indicate the presence of a trajectory in clinical research expertise. To deal with this issue, a peer learning training system has been implemented in an anesthesiology residency in the United States ^(17)^. Peer learning, in which trainees teach each other in small groups, provides trainees with a psychologically “safe” environment to learn and facilitate their deeper understanding of educational contents and reflections ^(18)^. This teaching and learning methodology can create a mutual mentorship environment for both young and experienced researchers. Administrators can also save human and funding resources by making arrangements for research instructors using this methodology. Therefore, implementing educational techniques, such as peer learning, for clinical research training can be effective in promoting the academic activities of pediatric field clinicians.

There are several limitations in this study. First, the response rate of this study was low, as the recruitment in this online survey was conducted by email, which means that there would be a selection bias for respondents in this study. Those who responded might be more interested in clinical research compared with those who did not. However, it is also possible to state that we could include the more highly motivated participants who would be the effective target of our future research support systems and that this selection bias may not lower the value of our findings. Second, although this survey was conducted using the JACHRI network involving 34 hospitals from broad areas within Japan, most of the participating 27 hospitals were tertiary children’s hospitals and university hospitals; thus, another selection bias could exist. The generalizability of our findings is therefore still unclear, and studies with larger sample size from more diverse institutions are needed to overcome these limitations. This study can serve as a pilot study for these future, hopefully well-designed studies.

In conclusion, Japanese physicians’ support requests for academic activities and clinical research were different, depending on the number of years after graduation and the level of experience in clinical research. In order to enhance clinical research and academic activities in pediatrics, it is important to provide appropriate types and levels of education and support programs.

## Article Information

### Conflicts of Interest

None

### Acknowledgement

We would like to express our gratitude to Dr. Takashi Igarashi, the president of the Japanese Association of Children’s Hospitals and Related Institutions (JACHRI), staff members of JACHRI, and its member hospitals, all of whom supported the present study. We would also like to thank all the people who responded to the questionnaire. In addition, we thank the medical editors at the National Center for Child Health and Development for their editorial/language assistance.

### Author Contributions

Osamu Nomura drafted the initial manuscript and approved the final version of the manuscript as submitted.

Toru Kobayashi initiated the research project, developed the questionnaire, collected the data, and approved the final version of the manuscript as submitted.

Chie Nagata conducted statistical analysis, critically reviewed the draft manuscript, and approved the final version of the manuscript as submitted.

Takeshi Kuriyama critically reviewed the draft manuscript and approved the final version of the manuscript as submitted.

Mayumi Sako critically reviewed the draft manuscript and approved the final version of the manuscript as submitted.

Kazuyuki Saito supervised the research project, critically reviewed the draft manuscript, and approved the final version of the manuscript as submitted.

Akira Ishiguro supervised the research project, contributed to the conception, critically reviewed the draft manuscript, and approved the final version of the manuscript as submitted.

### Approval by Institutional Review Board (IRB)

This study was approved by the Ethics Committee of the National Center for Child Health and Development (ID: 2159)
